# Subacute liver failure caused by hyperthyroidism and complicated by drugs: a case report and review of the literature

**DOI:** 10.3389/fmed.2025.1629957

**Published:** 2025-09-02

**Authors:** Yiwei Hou, Mingxu Tong, Yu Yang, Zijun Zhou, Yunxi Fu, Zhennian Gou, Jinting Xi, Xiangyu Liao, Shixin Li, Wufei Zhu

**Affiliations:** ^1^The First College of Clinical Medical Science, China Three Gorges University, Yichang, Hubei, China; ^2^Department of Endocrinology, Yichang Central People’s Hospital, Yichang, Hubei, China; ^3^Department of Hepatobiliary Surgery, Yichang Central People’s Hospital, Yichang, Hubei, China; ^4^Medical Technology College of Qiqihar Medical College, Qiqihar, Heilongjiang, China; ^5^Department of Oncology, Yichang Central People’s Hospital, Yichang, Hubei, China

**Keywords:** hyperthyroidism-induced liver damage, drug-induced liver injury, subacute liver failure, double plasma molecular adsorption system, case report

## Abstract

**Background:**

Severe liver dysfunction due to concurrent hyperthyroidism and drug-induced liver injury (DILI) is exceedingly rare, posing significant diagnostic and therapeutic challenges. This case report highlights the complexity of managing simultaneous hepatic injuries caused by hyperthyroidism itself and the hepatotoxic effects of antithyroid medications. Such dual etiologies necessitate careful clinical evaluation and innovative treatment approaches, including artificial liver sup89 port.

**Case summary:**

A 34-years-old male presented with progressive weight loss for 1 year and severe jaundice for 1 month. He had previously been diagnosed with hyperthyroidism and treated intermittently with methimazole and propranolol. Upon admission, laboratory evaluations revealed markedly elevated liver enzymes and bilirubin (ALT 255 U/L, AST 109 U/L, total bilirubin 697.55 μmol/L). His thyroid function was severely impaired (TSH 0.012 μIU/mL, FT3 > 30.8 pmol/L, FT4 > 154.8 pmol/L). The patient underwent multidisciplinary management, including glucocorticoids, plasma exchange, and bilirubin adsorption therapy. Despite multiple therapeutic interventions, his clinical status fluctuated significantly. The decision to discontinue antithyroid medications due to exacerbating liver injury complicated the hyperthyroidism management further. Eventually, treatment with radioactive iodine was successfully implemented, resulting in stabilized thyroid and liver function.

**Conclusion:**

Clinicians must consider overlapping etiologies of liver injury in hyperthyroid patients and employ multidisciplinary approaches for effective management.

## 1 Introduction

Hyperthyroidism is a common endocrine disorder characterized by excessive production and release of thyroid hormones, typically presenting with weight loss, palpitations, and fatigue. Hepatic dysfunction associated with hyperthyroidism, though recognized, is usually mild and self-limiting with appropriate management (1). Severe hepatic impairment, manifesting as acute hepatic dysfunction or failure due to hyperthyroidism or its treatments is rare, and the literature addressing such severe cases remains sparse (2). This report presents a unique and complex case of severe hepatic dysfunction and multi-organ involvement in a patient with hyperthyroidism exacerbated by abrupt discontinuation of antithyroid medication.

The primary purpose of this case report is to illustrate the clinical intricacies, diagnostic challenges, and management complexities in severe hyperthyroidism-associated hepatic injury. We conducted an extensive literature review using databases such as PubMed, Scopus, and Web of Science, employing search terms including “hyperthyroidism,” “thyrotoxicosis,” “hepatic dysfunction,” and “drug-induced liver injury.” This literature review underscores the rarity and complexity of severe hepatic outcomes in hyperthyroidism, further highlighting the uniqueness and clinical significance of this particular case.

This case involves a 34-years-old male presenting primarily with prolonged weight loss exceeding 1 year and progressive jaundice over 1 month, following abrupt cessation of antithyroid medications. Given the severe clinical manifestations, multidisciplinary management approaches were necessary, including artificial liver support and plasmapheresis. This report thus not only adds valuable insights into rare clinical presentations of hyperthyroidism-related hepatic dysfunction but also emphasizes the importance of cautious management strategies, detailed medication histories, and multidisciplinary interventions to achieve optimal clinical outcomes.

## 2 Case presentation

A 34-years-old male employed locally presented primarily with significant weight loss persisting over 1 year and progressive jaundice lasting for 1 month. His medical issues began insidiously in 2018 with unexplained gradual weight loss, which he initially disregarded, and consequently did not seek medical evaluation. By July 2019, symptoms had escalated to include palpitations, exertional dyspnea, fatigue, and increased appetite despite continuing weight loss. He was subsequently diagnosed at an endocrine clinic with hyperthyroidism complicated by hepatic dysfunction. Initial treatment comprised oral methimazole (10 mg three times daily), oral propranolol (10 mg three times daily), and unspecified hepatoprotective medications. Approximately 45 days before hospitalization for treatment, the patient independently discontinued the prescribed medications.

Following this abrupt cessation, the patient rapidly deteriorated, experiencing pronounced jaundice, nausea, frequent watery vomiting, anorexia, severe fatigue, exertional chest discomfort, profuse sweating, further weight loss, severe generalized pruritus, increased nocturia (4–5 times nightly with dark-colored urine), and frequent pale, oily stools (approximately 5 times daily). Additional symptoms included significantly disturbed sleep and bilateral lower limb edema. Notably, he reported no fever, chills, abdominal pain, respiratory distress, bleeding tendencies, or joint pain.

His past medical history was negative for chronic conditions such as hypertension, diabetes mellitus, cardiovascular diseases, hepatitis, tuberculosis, or any surgical interventions. He reported an allergy to dexamethasone characterized by severe muscular weakness and paralysis, but provided no exact dates or serum drug levels related to this reaction. Family history was negative for hereditary conditions, with both parents healthy. The patient maintained a dietary habit typical of the local region, with no unusual food intake reported. His social history was significant for tobacco use (one pack daily for 17 years) and negative for alcohol or illicit drug use.

On physical examination at admission, the patient was jaundiced with mild proptosis, typical hepatic facies, and bilateral lower limb edema but no spider angiomas or palmar erythema. Vital signs were stable with temperature 36.7 °C, heart rate 98 bpm, respiratory rate 22 breaths/min, and blood pressure 131/79 mmHg. Cardiopulmonary assessment was normal apart from mild tachycardia. The abdomen was soft, non-tender, without palpable hepatosplenomegaly or masses, and Murphy’s sign was negative.

Pertinent laboratory investigations revealed elevated liver enzymes [ALT 255 U/L (7–40 U/L), AST 109 U/L (13–35 U/L)], markedly increased TBIL [697.55 μmol/L (0–23.0 μmol/L)] and DBIL [472.25 μmol/L (0–6.8 μmol/L)], reduced albumin [30.67 g/L (40–55 g/L)], hypokalemia [3.36 mmol/L (3.5–5.3 mmol/L)], and mild hyponatremia [136.3 mmol/L (137.0–147.0 mmol/L)]. Severe hyperthyroidism was evident with suppressed TSH [0.012 μIU/mL (0.35–5.5 μIU/mL)], significantly elevated FT3 [>30.8 pmol/L (3.5–7.0 pmol/L)], and FT4 [>154.8 pmol/L (10.0–22.0 pmol/L)] (Table 1). Mild anemia was observed [Hb 115 g/L (120–160 g/L)], whereas white cell and platelet counts were within normal ranges.

**TABLE 1:** Sequential thyroid function test results during treatment.

Date	FT3 (pmol/L)	FT4 (pmol/L)	TSH (μ IU/mL)
Nov 29, 2019 (Day)	>30.8	>154	0.008
Feb 27, 2020 (Day 1)	>30.8	>154	0.012
Mar 12, 2020 (Day 15)	3.37	20.31	0.012
Mar 20, 2020 (Day 23)	2.41	11.47	0.026
Apr 9, 2020 (Day 43)	6.63	28.76	0.026

**FIGURE 1:**
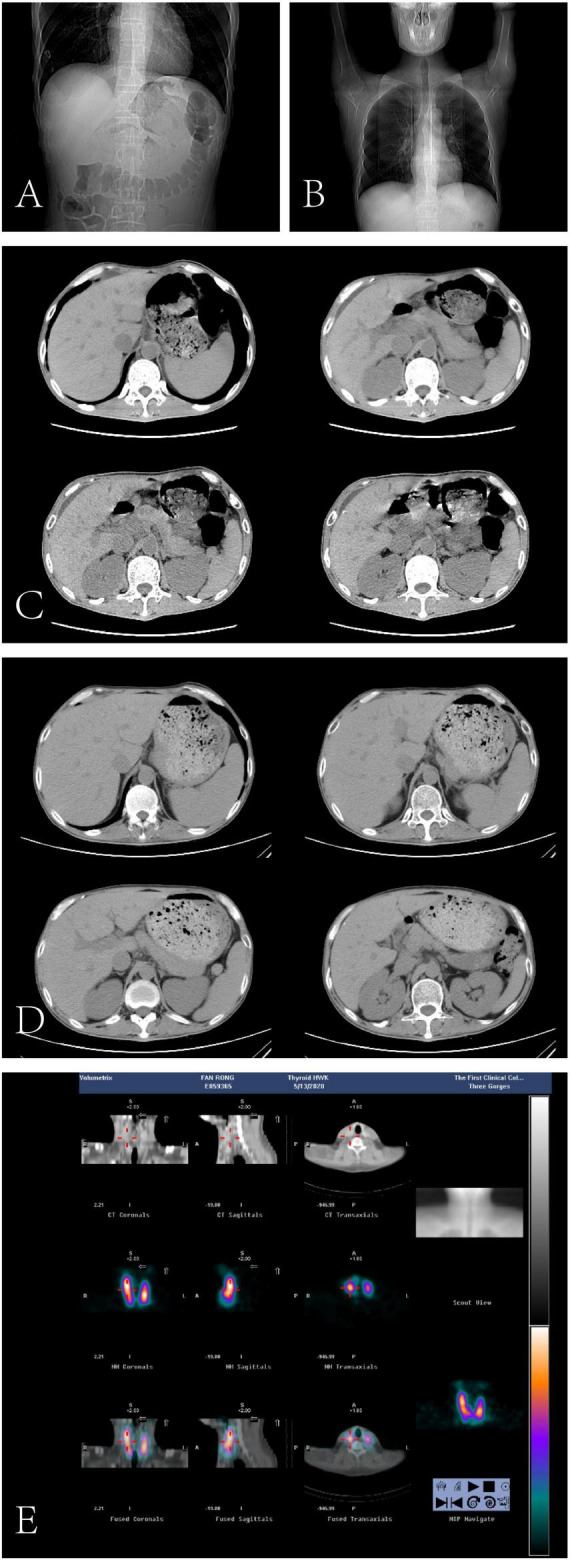
Imaging findings from February 27, 2020, to May 13, 2020. **(A,C)** (Non-contrast CT of the hepatobiliary system, pancreas, and spleen; 2020-02-27): The liver demonstrates preserved morphology with small nodular hyperdense lesions and patchy hypodense areas. Minimal perihepatic ascites is noted. The gallbladder exhibits irregular wall thickening with increased density, while the intra- and extrahepatic bile ducts remain non-dilated. A 1.4 cm iso-dense nodular lesion is observed at the splenic hilum. The pancreas appears unremarkable. Left renal calculi (multiple punctate hyperdensities) and diffuse bowel gas-fluid distention without obstruction are evident. **(B,D)** (Non-contrast CT of the hepatobiliary system, pancreas, spleen, and chest; 2020-04-04): Follow-up imaging reveals resolution of perihepatic ascites and improvement in bowel gas-fluid distention compared to prior study (2020-02-27). Pulmonary findings include increased lung markings with scattered linear and punctate opacities in both lungs, suggesting mild inflammatory or fibrotic changes. The thyroid gland shows reduced parenchymal density. Persistent features include hepatic nodular hyperdensities, gallbladder wall thickening, the splenic hilar lesion, and left renal calculi. **(E)** (Planar thyroid scintigraphy with Technetium-99m pertechnetate; 2020-05-13): Marked thyroid enlargement is evident with preserved technetium uptake, indicating a non-functionally impaired gland. Clinical correlation is advised to evaluate etiology (e.g., goiter, thyroiditis).

Imaging studies provided additional diagnostic clarity. High-resolution chest CT indicated minor fibrotic lesions in both lungs, without active infiltrates, and decreased thyroid density. Abdominal and pelvic CT scans showed hepatic calcifications, small liver cysts, thickened gallbladder walls indicative of chronic cholecystitis, minimal ascites, and left renal calculi. Ultrasound demonstrated diffuse thyroid enlargement with heterogeneous echotexture and mixed echogenic nodules (Figure 1).

**FIGURE 2:**
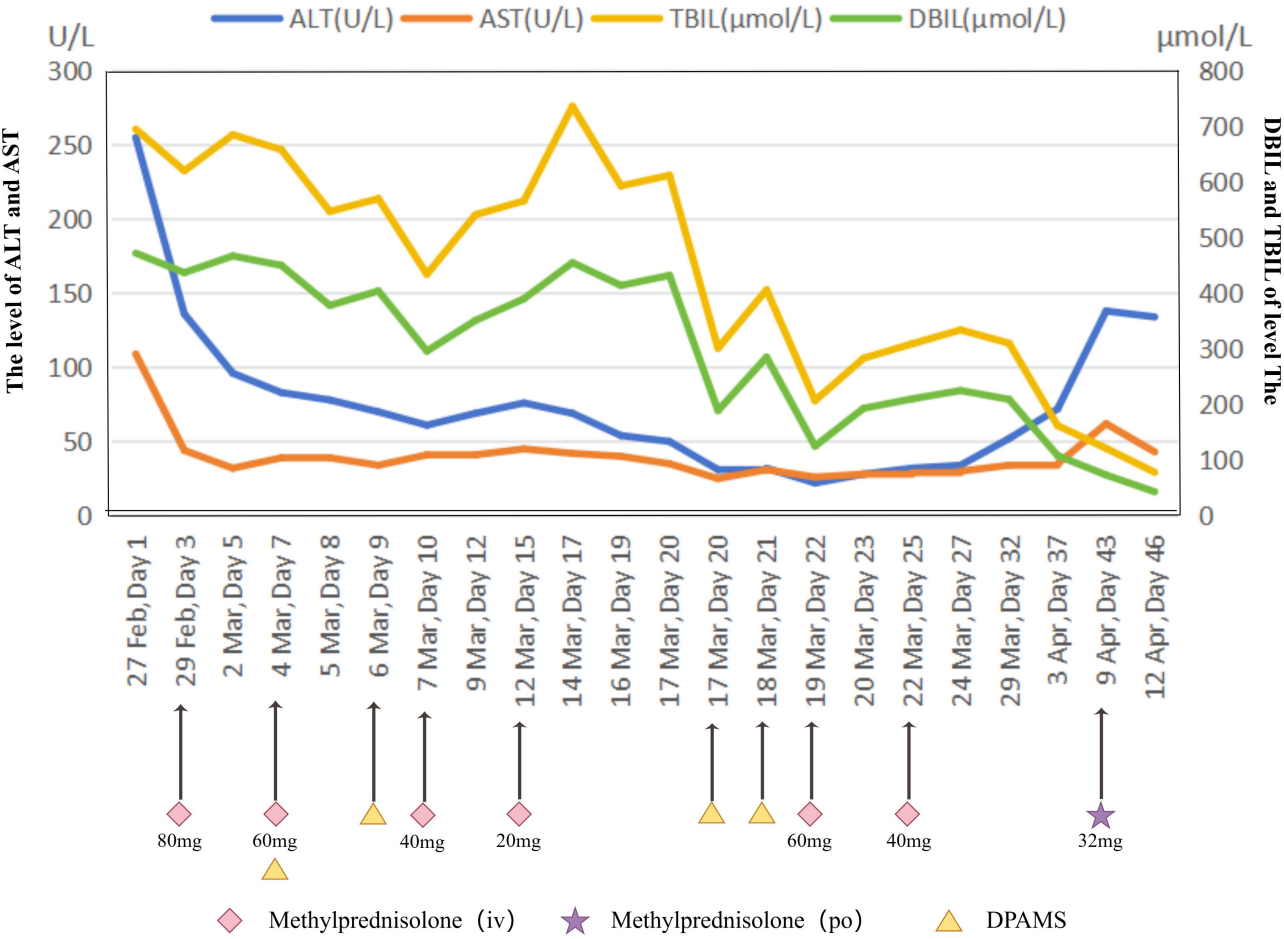
The dynamic evolution of biochemical indicators of a patient under hormone and DPMAS treatments (February 27 - April 12, 2020). This line graph shows the dynamic changes of several biochemical indexes in a patient during the treatment period from February 27 to April 12, 2020. The abscissa represents the treatment time, and the ordinate on the left shows the values of liver function indexes ALT and AST, while the ordinate on the right shows the values of TBIL and DBIL. Lines of different colors represent different indices. Methylprednisolone was used for hormone treatment during the treatment period, including two administration routes: intravenous injection (IV, marked by pink diamonds) and oral administration (PO, marked by purple pentagrams), with different dosages at different time points. Meanwhile, DPMAS treatment (marked by yellow triangles) was also carried out. These treatment measures are interrelated with the fluctuations of various biochemical indexes, reflecting the dynamic response of the patient’s physical indexes during the treatment process.

**FIGURE 3:**
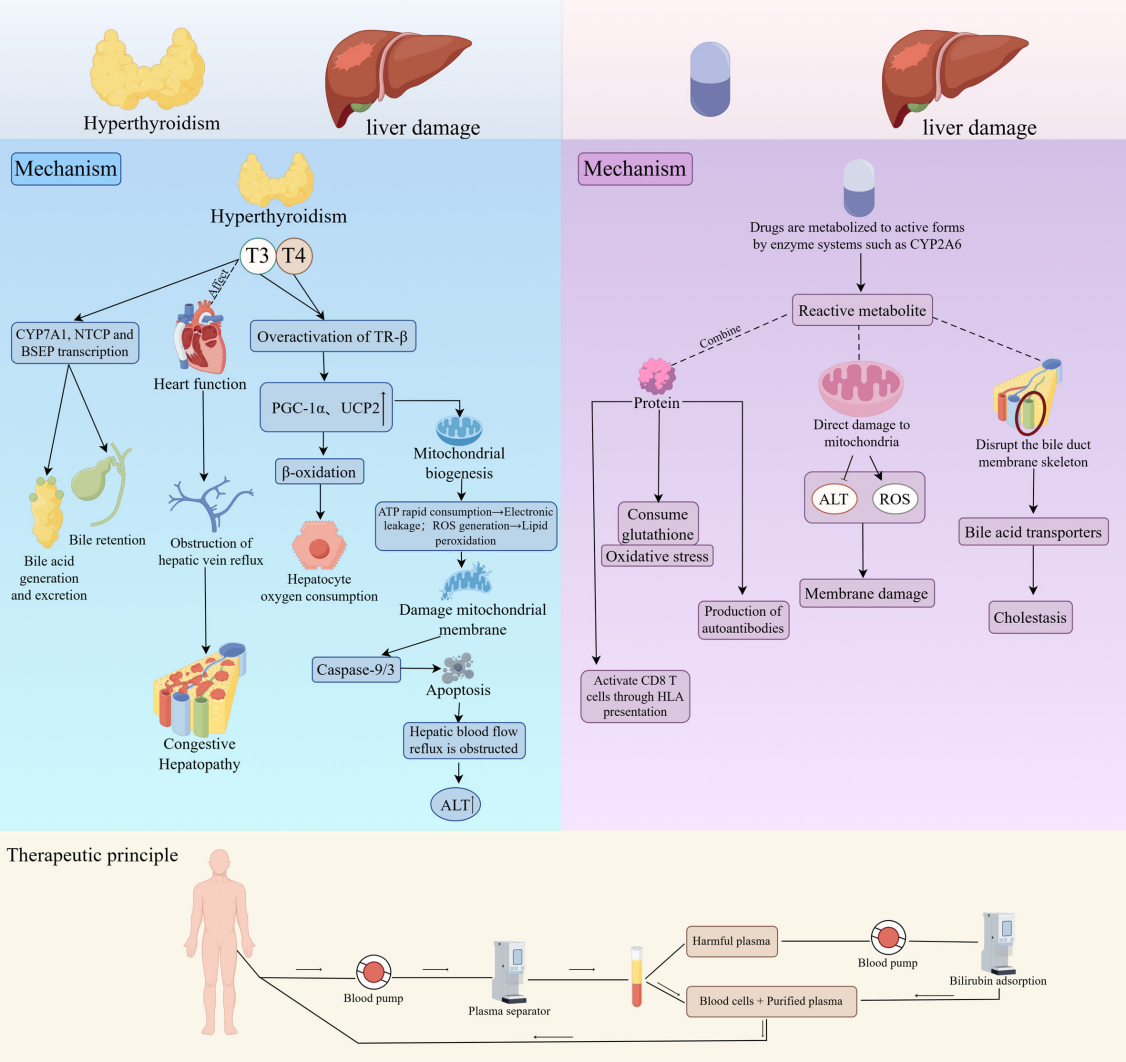
Mechanisms of liver damage induced by hyperthyroidism and drugs and treatment principles. This figure illustrates the mechanisms of liver damage caused by hyperthyroidism and drugs, as well as the treatment principles. Mechanism of liver damage caused by hyperthyroidism (left-hand side area): In hyperthyroidism, thyroid hormones T3 and T4 over-activate TR-β, triggering a series of reactions. By upregulating the transcription of CYP7A1, NTCP, and BSEP, it affects the production and excretion of bile acids, leading to cholestasis. Activation of PGC-1α and UCP2 enhances β-oxidation, inducing mitochondrial biogenesis, resulting in excessive ATP consumption and electron leakage, generating reactive oxygen species (ROS), causing lipid peroxidation, damaging the mitochondrial membrane, activating Caspase-9/3, and inducing hepatocyte apoptosis. It also affects heart function, obstructs hepatic vein reflux, increases hepatocyte oxygen consumption, and causes congestive hepatopathy, ultimately leading to an increase in alanine aminotransferase (ALT). Mechanism of liver damage caused by drugs (right-hand side area): Drugs are metabolized into reactive metabolites by enzyme systems such as CYP2A6. These metabolites can bind to proteins, deplete glutathione, trigger oxidative stress, generate autoantibodies, and activate CD8 + T cells through HLA presentation. They can also directly damage mitochondria, increase ALT levels, generate ROS, disrupt the biliary duct membrane skeleton, and affect bile acid transporters, leading to cholestasis and membrane damage. Treatment principle (bottom-hand side area): Blood is pumped out of the body through a blood pump, separated by a plasma separator, and harmful plasma is removed. Blood cells are then reinfused with the purified plasma. Additionally, bilirubin adsorption can be used to purify the blood further, aiming to treat liver damage.

The patient presented a complex diagnostic scenario characterized by overlapping clinical manifestations. Specifically, the negative viral serologies for hepatitis A, B, C, and E viruses, as well as cytomegalovirus, Epstein–Barr virus, and herpes simplex virus, rule out acute or chronic viral hepatitis. The absence of autoantibodies (ANA, SMA, LKM-1) and normal immunoglobulin G levels makes autoimmune hepatitis highly unlikely. Normal copper metabolism and ceruloplasmin levels, together with the absence of Kayser–Fleischer rings, exclude Wilson’s disease. Iron studies within normal limits effectively rule out hereditary hemochromatosis. The lack of alcohol consumption, metabolic risk factors, and imaging evidence of steatosis excludes alcoholic and non-alcoholic fatty liver disease. Normal imaging without biliary dilatation rules out extrahepatic cholestasis, and the absence of hemodynamic instability or cardiac dysfunction excludes ischemic hepatitis and congestive hepatopathy. Finally, a thorough medication and herbal supplement review revealed no alternative hepatotoxic agent, which suggest hyperthyroidism-induced liver injury, drug-induced hepatotoxicity, and acute hepatic dysfunction. Recurrent elevations in bilirubin and fluctuations in hepatic function presented significant therapeutic challenges (Figure 2). The patient received multidisciplinary care involving hepatology, endocrinology, and intensive supportive therapies, including plasma exchange, artificial liver support, corticosteroids, broad-spectrum antibiotics, hepatoprotective treatments, electrolyte replacement, and antithyroid therapy. Clinical outcomes demonstrated gradual improvement, evidenced by normalization of thyroid function, improvement in hepatic function, and resolution of jaundice and electrolyte disturbances (Figure 3 and Table 2).

**TABLE 2:** Published case reports (2017–2024) of thyroid disease complicated by antithyroid drug-induced liver injury.

Year	Reference (first author et al., title, journal)	Study type	Key findings
2024	Deng L et al. “Apathetic Graves’ disease with severe hepatic and renal dysfunction induced by COVID-19 infection.” *Medicine (Baltimore)* 103(11): e37456 (17).	Case report + literature review	COVID-19 may precipitate apathetic Graves’ disease with simultaneous severe hepatotoxicity and renal impairment. Multidisciplinary management plus individualized low-dose methimazole or early 131I therapy normalized thyroid and liver function within 4 months while preserving renal status.
2017	Ji H et al. “A rare case of methimazole-induced cholestatic jaundice in an elderly Asian man with hyperthyroidism.” *Medicine (Baltimore)* 96(49): e9093 (18).	Case report	Cholestatic jaundice developed shortly after methimazole initiation. Liver enzymes improved on drug withdrawal. Roussel-Uclaf causality scoring underscores an ethnicity-linked hepatotoxicity risk that clinicians should monitor in Asian patients.
2020	Nagarajan VD, Morales A, Pleasant L, Shenoi A. “Sepsis and thyroid storm in a patient with methimazole-induced agranulocytosis.” BMJ Case Rep 13(7): e235536. (19).	Case report	A 15-years-old female patient with Graves’ disease developed agranulocytosis after taking methimazole, followed by sepsis and thyroid storm. Due to contraindications to first-line antithyroid drugs, plasmapheresis was used to control the thyroid storm as a bridging therapy, and thyroidectomy was eventually performed. This pediatric case is the first to confirm the value of plasmapheresis in such complex thyroid, emphasizing the need for early identification of agranulocytosis and urgent intervention.

The patient was admitted for continuous inpatient management over a period of 40 days, during which all diagnostic evaluations, therapeutic interventions, and clinical monitoring were conducted within the hospital setting, ensuring uninterrupted multidisciplinary care and timely adjustment of treatment strategies according to dynamic changes in his hepatic and thyroid function.

Following completion of a 40-days course of continuous inpatient management and stabilization of hepatic function, the patient was discharged in a clinically improved condition. Thereafter, he underwent outpatient radioactive I131 therapy at a dose of 15 mCi, which achieved sustained control of hyperthyroidism without precipitating any further hepatic decompensation.

At post-discharge follow-up after I131 therapy, thyroid function testing demonstrated FT3 6.01 pmol/L, FT4 24.42 pmol/L, TSH 0.014 μIU/mL, TRAb 24.60 U/L, with negative TPOAb, while liver function tests showed ALT 46 U/L, AST 15 U/L, TBIL 22.86 μmol/L, DBIL 8.27 μmol/L. During subsequent outpatient follow-up visits on June 15, 2020, August 14, 2020, November 10, 2020, December 10, 2020, and February 9, 2021, thyroid function, liver function, and complete blood counts remained within normal limits, indicating sustained biochemical remission and favorable clinical outcome.

The detailed clinical chronology established a causal relationship between hyperthyroidism, medication discontinuation, herbal medicine consumption, and severe hepatic dysfunction, underscoring the necessity for thorough medication oversight and prompt multidisciplinary intervention.

## 3 Discussion

This case presented a complex clinical scenario involving a 34-years-old male with severe hyperthyroidism accompanied by significant hepatic dysfunction manifesting as progressive jaundice, marked weight loss, and multi-organ system involvement. The patient’s presentation aligns well with reported literature on hyperthyroidism-induced hepatic injury, particularly the combination of elevated liver enzymes, severe hyperbilirubinemia, and profound metabolic derangements (3). However, several unique elements distinguish this case from typical presentations in existing literature.

Firstly, the patient’s autonomous discontinuation of antithyroid medications complicates the clinical picture. Literature consistently notes hepatotoxicity associated with antithyroid drugs, making the attribution of causality complex (4). According to previous studies, methimazole-induced hepatic injury is relatively uncommon but can manifest with severe cholestatic or hepatocellular patterns (5). Thus, it remains challenging to clearly distinguish between hyperthyroidism-induced liver dysfunction and drug-induced liver injury (DILI) in this patient (6).

The temporal relationship observed supports a causal link between medication withdrawal and rapid hepatic decompensation. The patient’s rapid deterioration post-medication discontinuation strongly suggests medication-related hepatotoxicity (7). Application of the Naranjo probability scale to this case. The Naranjo probability scale suggests a “probable” drug-induced liver injury (DILI) due to methimazole, influenced by concurrent hyperthyroidism. Alternative causes were excluded, and the liver dysfunction appeared after methimazole discontinuation, aligning with the temporal relationship criteria. Although hyperthyroidism itself can contribute to liver injury, the severity and rapid progression of hyperthyroidism-induced liver injury is mild, suggesting methimazole played a significant role. The patient’s recovery after treatment further strengthens this association, despite no re-challenge being performed. The final score classifies the injury as “probable” DILI, underscoring the importance of detailed medication histories in clinical assessments (5).

Comparatively, existing literature typically describes milder hepatic complications in hyperthyroid patients, often resolving quickly with appropriate antithyroid medication adjustments or discontinuation (8). This patient’s case diverges markedly from this trend, presenting an unusually severe hepatic dysfunction requiring intensive interventions, including artificial liver support and plasma exchange, which highlights the necessity for prompt identification and multidisciplinary management in such rare, severe cases.

Limitations of this case report include the absence of drug concentration data and the lack of definitive biopsy confirmation of the hepatic pathology. These limitations constrain definitive causal attribution and limit generalizability. Nevertheless, detailed clinical, biochemical, and imaging documentation strengthens the accuracy and validity of this case.

Despite these limitations, the literature supports the comprehensive multidisciplinary interventions provided, particularly artificial liver support systems, plasmapheresis, intensive hepatoprotective therapies, and tailored endocrine management (9–12). Literature evidence confirms the benefit of early aggressive management in similar severe hepatic complications, reinforcing the validity of the treatment decisions undertaken here (13, 14).

In conclusion, this case underscores the clinical complexity and diagnostic challenges associated with hyperthyroidism-induced hepatic dysfunction compounded by drug-induced injury (15). The primary lessons from this report include the necessity of thorough medication reconciliation and the critical role of multidisciplinary management approaches in achieving favorable outcomes in complicated presentations of thyroid and hepatic dysfunction (16).

## 4 Conclusion

This case highlights the clinical significance and diagnostic complexity of severe hepatic dysfunction associated with hyperthyroidism and complicated by drug-induced liver injury. It underscores the critical importance of detailed medication reconciliation, including non-prescription remedies, to promptly identify and manage potential hepatotoxic risks. Evidence-based recommendations derived from this case include rigorous monitoring of liver function tests during antithyroid medication therapy and early implementation of multidisciplinary treatment approaches involving hepatology, endocrinology, and intensive care. Integrating these practices into clinical routine will enhance patient safety and outcomes. Further research is warranted to clarify the hepatotoxic potential of specific medicines and to establish more precise guidelines for the management of hyperthyroidism-associated liver injury.

## Data Availability

The original contributions presented in this study are included in this article/supplementary material, further inquiries can be directed to the corresponding author.
